# A Complex of Pyrosequencing-Based Methods for Detection of Somatic Mutations in Codons 600 and 601 of the *BRAF* gene

**DOI:** 10.17691/stm2022.14.2.04

**Published:** 2022-03-28

**Authors:** O.P. Dribnokhodova, A.S. Esman, V.I. Korchagin, A.Yu. Bukharina, E.A. Dunaeva, G.V. Leshkina, E.V. Borisova, Ya.A. Voiciehovskaya, A.I. Daoud, V.N. Khlyavich, K.O. Mironov

**Affiliations:** Senior Researcher, Scientific Group for Development of New Methods of Genetic Polymorphisms Detection; Central Research Institute of Epidemiology of the Federal Service for Surveillance on Consumer Rights Protection and Human Wellbeing, 3A Novogireevskaya St., Moscow, 111123, Russia;; Researcher, Scientific Group for Development of New Methods of Genetic Polymorphisms Detections; Central Research Institute of Epidemiology of the Federal Service for Surveillance on Consumer Rights Protection and Human Wellbeing, 3A Novogireevskaya St., Moscow, 111123, Russia;; Researcher, Scientific Group for Development of New Methods of Genetic Polymorphisms Detections; Central Research Institute of Epidemiology of the Federal Service for Surveillance on Consumer Rights Protection and Human Wellbeing, 3A Novogireevskaya St., Moscow, 111123, Russia;; Junior Researcher, Scientific Group for Genomics and Post-genome Technology; Central Research Institute of Epidemiology of the Federal Service for Surveillance on Consumer Rights Protection and Human Wellbeing, 3A Novogireevskaya St., Moscow, 111123, Russia;; Researcher, Scientific Group for Development of New Methods of Genetic Polymorphisms Detections; Central Research Institute of Epidemiology of the Federal Service for Surveillance on Consumer Rights Protection and Human Wellbeing, 3A Novogireevskaya St., Moscow, 111123, Russia;; Biologist, Molecular Diagnostic Methods Department; Central Research Institute of Epidemiology of the Federal Service for Surveillance on Consumer Rights Protection and Human Wellbeing, 3A Novogireevskaya St., Moscow, 111123, Russia;; Physician, Clinical Diagnostic Laboratory; Central Research Institute of Epidemiology of the Federal Service for Surveillance on Consumer Rights Protection and Human Wellbeing, 3A Novogireevskaya St., Moscow, 111123, Russia;; Researcher, Scientific Group of Genetic Engineering and Biotechnology; Central Research Institute of Epidemiology of the Federal Service for Surveillance on Consumer Rights Protection and Human Wellbeing, 3A Novogireevskaya St., Moscow, 111123, Russia;; Head of Clinical Diagnostic Laboratory; Foreign Unitary Consultancy Enterprise MedArt, 1B Platonov St., Minsk, 220034, Republic of Belarus; Ultrasound Diagnostic Physician, Oncologist; Foreign Unitary Consultancy Enterprise MedArt, 1B Platonov St., Minsk, 220034, Republic of Belarus; Head of Scientific Group for Development of New Methods of Genetic Polymorphisms Detection; Central Research Institute of Epidemiology of the Federal Service for Surveillance on Consumer Rights Protection and Human Wellbeing, 3A Novogireevskaya St., Moscow, 111123, Russia;

**Keywords:** pyrosequencing, *BRAF*, oncogenetics, fine-needle aspiration biopsy, thyroid cancer

## Abstract

**The aim of the study** is to develop methods for the differentiation of mutations in the *BRAF* codon 600 and to increase the sensitivity of the K601E mutation detection.

**Materials and Methods:**

The nucleotide sequence of the *BRAF* codons 592–602 was identified using the PyroMark Q24 genetic analysis system. The mutations search in codon 600 was conducted using the 600-S primer in line with the following order of adding nucleotides: GCTGTCАTCTGCTAGCTAGAC (corresponding to nucleotides 1799–1786). The K601E mutation was detected using the 601-S primer in line with the following order of nucleotide addition: GCTACTCACTGTAG (corresponding to nucleotides 1801–1793). The analytical characteristics of the proposed methods for somatic mutations’ detection were determined using dilutions of plasmid DNA samples containing the *BRAF* gene region without mutations or with one of the following mutations: V600E, V600R, V600K, V600M, and K601E. Validation was performed on 132 samples of biological material obtained from the thyroid nodules.

**Results:**

The developed methods allow to determine 2% of the V600E or V600M mutations, 1% of the V600K and V600R mutations, and 3% of the K601E mutations in samples with high DNA concentration; it is also possible to confidently detect at least 5% of the mutant allele for all mutations in low concentration samples (less than 500 copies/PCR). During biological material testing, 53 samples with the V600E mutation were detected; the proportion of the mutant allele was 4.9–50.0%.

**Conclusion:**

A complex of methods for determination of the nucleotide sequence of the *BRAF* codons 592–601 and the algorithm for testing samples and analyzing mutations in the *BRAF* codons 600–601 was developed. The method provides sufficient sensitivity to detect frequent mutations in codons 600 and 601 and allows them to be precisely differentiated.

## Introduction

The *BRAF* (v-Raf murine sarcoma viral oncogene homolog B) gene encodes serine/threonine protein kinase, which is a part of the MAPK/ERK signaling pathway, the constitutive activation of which results in oncogenic transformation of cells. Activating somatic mutations in the *BRAF* gene are detected in 6–8% of cases of solid tumors [1, 2], including melanoma (in 44% of tumors), thyroid cancer (1.7–90.0%, depending on the tumor histological type), colorectal adenocarcinoma (10%), and lung adenocarcinoma (1.5–8.0%) [1, 3, 4].

The proportion of the most frequent *BRAF* mutation c.1799 T>A p.V600E significantly varies within the range of values below 10% in bladder cancer to over 90% in thyroid cancer [3, 4]. For a number of nosologies, it has been shown that tumors with various *BRAF* mutations differ in clinical characteristics, clinical course, treatment response, and prognosis [1, 2, 4].

The presence of the *BRAF* mutations is a predictive marker for response to treatment with target therapy aimed at the MAPK/ERK signaling pathway [1, 2, 5, 6]. However, for tumors with non-V600E and especially non-V600 mutations, the effectiveness of the *BRAF* inhibitors is lower [2, 7].

Tumors with the *BRAF* mutations often demonstrate a more aggressive clinical course, as it was seen in melanoma and thyroid cancer. In colorectal cancer, the V600E mutation is associated with lower overall survival and progression-free survival compared with *BRAF* wild-type tumors, whereas the overall survival median for tumors with non-V600E mutations is higher than with the V600E mutation [1, 6]. Data on the prognostic impact of the *BRAF* mutations in lung cancer are controversial. This may be a result of the large proportion and diversity of mutations other than V600E, which are not taken into account in every study [1, 7].

In thyroid tumors, the V600E mutation is typical for papillary thyroid cancer, whereas K601E is typical for follicular neoplasms [8], which allows their detection to be used to clarify the diagnosis in such cytological findings as “atypia of undetermined significance” and “follicular tumor/suspicious for a follicular tumor” (III and IV diagnostic categories of the Bethesda system for reporting thyroid cytopathology classification, 2017 [9]) for thyroid nodules, as well as for the treatment choice [8, 10, 11]. The V600E mutation, especially in combination with mutations in the *TERT* gene promoter, is associated with extra-thyroidal extension, a more aggressive phenotype, and a high risk of recurrence [1, 8, 10, 11].

Therefore, when searching for mutations in the *BRAF* gene, it is reasonable to use methods that allow the detection and differentiation of clinically significant mutations in the presence of intact DNA. Currently, this is achieved by the real-time PCR and immunohistochemistry methods, which are characterized by high sensitivity and a relatively low cost. However, they can only be used to determine a limited range of mutations and are not always specific to the mutation type. Sequencing-based methods allow the detection and differentiation of the already known and new mutations [12-14]. Pyrosequencing is superior to Sanger sequencing in terms of sensitivity in the detection of a minor DNA fraction (about 15–20% for Sanger sequencing and 1–5% for pyrosequencing) [12-16]. Compared to high-throughput sequencing, pyrosequencing requires less analysis time and lower reagent costs [12-14]. Selection of optimal analysis parameters for pyrosequencing ensures high sensitivity and specificity in the detection of various mutations, as well quantitative measurement of the mutant allele fraction [15-18].

The Central Research Institute of Epidemiology of the Federal Service for Surveillance on Consumer Rights Protection and Human Wellbeing (Moscow, Russia) has earlier developed a method for determination of the *BRAF* nucleotide sequence of codons 592–602 and detection of all clinically significant mutations in this region. The detection limit was 2% for V600R and V600K, 3% for V600E and V600M, and 10% for K601E. However, at a mutation rate below 7–10%, it was difficult to precisely determine the mutation type in codon 600 [16].

**The aim of the study** is to develop methods for the differentiation of mutations in the *BRAF* codon 600 and to increase the sensitivity of the K601E mutation detection.

## Materials and Methods

### Pyrosequencing methods

The mutations were detected and quantitatively analyzed by determination of the nucleotide sequence by means of pyrosequencing using the PyroMark Q24 device (QIAGEN, Germany) [16, 17] with 5’biotin-gCT-TgC-TCT-gAT-Agg-AAA-ATg-AgA-TC3’ and 5’CCA-CAA-AAT-ggA-TCC-AgA-CAA-CT3’ amplification primers (fragment length is 172 base pairs) and with BR-S 5’gAC-CCA-CTC-CAT-CgA3’, 600-S 5’CCC-ACT-CCA-TCg-AgA-TTT-C3’ and 601-S 5’gAC-CCA-CTC-CAT-CgA-gAT-T3’ sequencing primers. Sequencing was performed in the reverse direction. The results were analyzed using the device software version 2.0.6.

**Figure 1.**
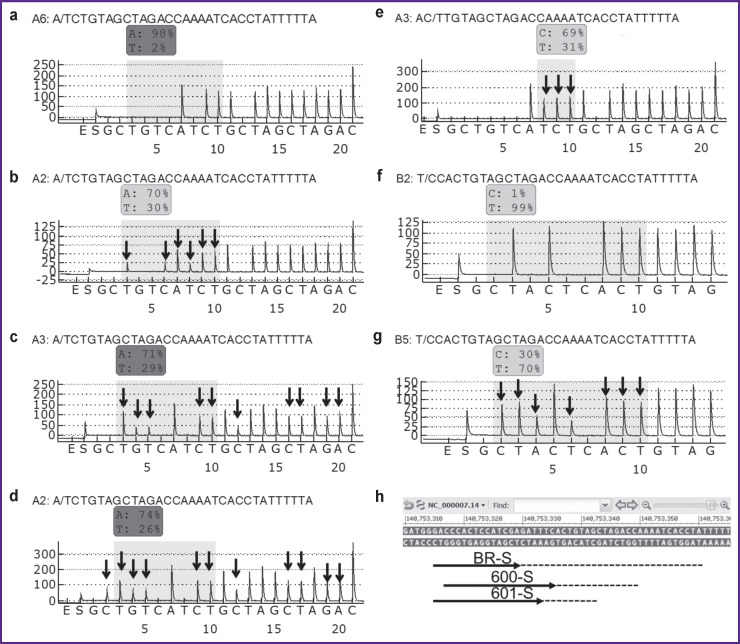
Sequenced region and examples of sample pyrograms: (а) a wild sample, which was sequenced using the 600-S primer; (b) a sample with the c.1799 T>A p.V600E mutation, 30% of the mutant allele, 600-S; (c) a sample with the c.1798_1799delinsAA p.V600K mutation, 30% of the mutant allele, 600-S; (d) a sample with the c.1798_1799delinsAG p.V600R mutation, 30% of the mutant allele, 600-S; (e) a sample with the c.1798 G>A p.V600M mutation, 30% of the mutant allele, 600-S; (f) a wild sample, sequenced using the 601-S primer; (g) a sample with the c.1801 A>G p.K601E mutation, 30% of the mutant allele, 601-S. The X-axis is the sequence of nucleotides supply into the reaction mixture; the Y-axis is the signal level detected by the device. The nucleotide sequences used for mutation analysis are shown above the pyrograms. The arrows indicate signals for nucleotides with the values changing in case of a mutation. (h) shows the arrangement of methods for the *BRAF* pyrosequencing: the reference sequence is NC_000007.14, the arrows indicate sequencing primers, the dotted line shows sequenced regions

Amplification, sample preparation, and pyrosequencing were performed according to the previously described method using reagents produced by the Central Research Institute of Epidemiology of the Federal Service for Surveillance on Consumer Rights Protection and Human Wellbeing (Russia) — AmpliSens — and QIAGEN (Germany) [18, 19]. Sequencing to determine the nucleotide sequence of codons 592–602 corresponding to nucleotides 1805–1775 (140753330-140753361 according to the reference sequence NC_000007.14) was conducted using the BR-S primer as stipulated in [16]. The mutations were searched in codon 600 using the 600-S primer in line with the following order of nucleotides addition: GCTGTCАTCTGCTAGCTAGAC (corresponding to nucleotides 1799–1786). The K601E mutation was detected using the 601-S primer in line with the following order of nucleotides addition: GCTACTCACTGTAG (corresponding to nucleotides 1801–1793) (Figure 1).

The type and proportion of the mutant allele were determined using the AQ Analyze function of the device software. The ratio of the mutant allele for mutations V600K and V600R, the nucleotide sequences of which are not suitable for automatic analysis, was calculated from the signal peaks on the pyrogram by the following formulas:

V600K=(1/3⋅G3+G4+T5+C12)/4;V600R=(C2+1/2⋅G3+G4+T5+C12)/5,

where C2, G3, G4, T5, C12 are the ratios of the corresponding signal level on the pyrogram (see Figure 1) to the average signal level.

### Analytical characteristics of the methods

The analytical characteristics were assessed through the following parameters: limit of blank — LOB (highest signal expected to be found when a blank sample containing no analyte are tested) and limit of detection — LOD (lowest analyte concentration likely to be reliable distinguished from the LOB value) [20]:

LOB=M+1.645σ,

where M and σ are the mean and standard deviations of the signal values in a batch of wild samples, respectively;

LOD=LOB+1.645σ,

where σ is the standard deviation of the signal values in a batch of samples with a mutation.

The analytical characteristics of the developed methods were determined on dilutions of the plasmid DNA samples containing the *BRAF* region cloned into the pGem-T vector, wild or having one of the following mutations: c.1799 T>A p.V600E; c.1798_1799delinsAG p.V600R; c.1798_1799delinsAA p.V600K; c.1798 G>A p.V600M; c.1801 A>G p.K601E. Mutagenesis was conducted by using the QuikChange II Site-Directed Mutagenesis Kit (Agilent Technologies, USA). The clone concentration was measured by real-time PCR with primers to the vector sequence. Each mutation was analyzed by mixtures containing 1, 2, 3, 5, 10, and 30% of the mutant allele. Mixtures with 1–5% of the mutant allele were tested in at least three replicates, 10 and 30% in two replicates for two DNA concentrations (100 and 10,000 copies/PCR) on two devices. A cloned wild-type *BRAF* sequence fragment of the same concentration was used as a control in each test.

### Biological samples

Validation of the methods was conducted on 132 samples of thyroid nodules taken from 127 patients. Of these, 131 samples were obtained by fine-needle aspiration biopsy (FNA) (needle washes after FNA in TE-buffer — 43; FNA cell materials were collected with a sterile scalpel on glass by traditional cytological method and stained according to Romanowsky method — 85; FNA samples placed into liquid preservative medium BD SurePath Collection Vial (Becton Dickinson, USA) — 3), from sections of paraffin blocks — 1. In addition to punctures of thyroid formations, FNA samples from the lymph nodes were obtained from four patients, whereas one patient had FNA samples taken simultaneously from the nodules in both thyroid lobes. At the time of testing completion, 57 patients had their histological diagnosis set. DNA was exported using RIBO-prep kits (Central Research Institute of Epidemiology of the Federal Service for Surveillance on Consumer Rights Protection and Human Wellbeing) and QIAamp DNA FFPE Tissue Kit (QIAGEN). The concentration of the extracted DNA was determined by real-time PCR using primers for the β-globin gene. Samples with low concentration (<500 copies/μl) were analyzed in several replications. All samples were tested using the BR-S primer to scan the entire region for mutations. Samples in which no mutations were found were sequenced using the 601-S primer to search for the K601E mutation. Samples with a mutation at codon 600 with less than 15% mutant allele were sequenced using the 600-S primer. Some samples were sequenced using three primers to compare the results. Along with biological samples, the authors analyzed a control sample of human DNA obtained from wild peripheral blood cells for each setup.

### Statistical data processing

Microsoft Excel was used for data preprocessing, tables organization and analysis, calculation of the main analytical indicators (LOB, LOD), and graph plotting. Embedded functionalities and add-ons of the R operating environment (https://www.R-project.org/) were used for statistical processing including distribution analysis, groups characteristics and intergroup differences, and statistical indicators calculations. Categorical data were evaluated using contingency tables, Pearson’s χ^2^ test, and Fisher’s exact test. The analysis of quantitative parameters, characterized by non-normal distribution (determined using the Shapiro–Wilk test and quantile-quantile (Q-Q) graphs plotting), outlying cases and insignificant sample size, was conducted using the following non-parametric tests: the Mann–Whitney test, the Dunn’s test, and the Wilcoxon paired test (for replicated observations analysis). The Bonferroni correction was used to correct for multiple comparisons. The test results were considered statistically significant with probability values (p-values) of type I error equal to p<0.05.

## Results

### Analytical characteristics of the methods

LOB determination was conducted using dilutions of the cloned wild-type *BRAF* sequence fragment in the amount of 100 and 10,000 copies/PCR (45 replicates with the 600-S primer for the V600E, V600K, V600M, and V600R mutations; 63 replicates with the 601-S primer for the K601E mutation), and also using human genomic DNA samples (18 and 29 replicates, respectively) isolated from peripheral blood cells in the amount of approximately 4000 copies/PCR.

The mutation load characteristics obtained after the analysis of the wild-type samples are shown in Table 1. The V600E, V600K, V600R, and K601E mutations demonstrate a deviation from the normal distribution (according to the results of the Shapiro–Wilk test and quantile-quantile graphs plotting), but the overall data are characterized by single outlying cases, whereas the mean values of the measured mutant allele fraction coincide with the medians.

**Table 1:** Fractions of the mutant allele in wild-type samples

Mutation	n	M±σ	Me [Q25; Q75]	Shapiro–(p) Wilk test
V600E	63	1.43±0.44	1.4 [1.1; 1.6]	0.0096
V600K	63	0.95±0.26	0.9 [0.7; 1.2]	0.0162
V600M	63	0.65±0.24	0.7 [0.4; 0.8]	0.0578
V600R	63	1.02±0.29	1.0 [0.8; 1.2]	0.0009
K601E	92	1.43±0.37	1.4 [1.2; 1.6]	0.0016

Note: n is the amount of samples analyzed.

The LOB level for mutations, calculated on the basis of the data received, ranged from 1.0 to 2.1% (Table 2).

**Table 2:** LOB and LOD values specified using the 600-S and 601-S primers

Mutation	LOB (%)	LOD, 10,000 copies/PCR (%)
V600E	2.1	3.0
V600M	1.0	1.3
V600K	1.4	1.7
V600R	1.5	2.0
K601Е	2.0	2.4

The LOD values were determined using a panel of the cloned controls dilutions with the 600-S primer for the V600E, V600K, V600M, and V600R mutations, and with the 601-S primer for the K601E mutation. The detected mutant allele fractions in the group of samples with a dilution of 100 copies/PCR had a high coefficient of variation (CV) (Table 3): for a mutation fraction of 1–5%, the mean CV (M_CV_) amounted to 45.53±19.25, for a mutation fraction of 30% — to 20.12±16.79. At a dilution of 10,000 copies/PCR for samples with the mutant allele fraction of 1–5%, M_CV_ amounted to 9.60±4.91, and for a mutation fraction of 30% — to 3.18±1.23. The comparison of the mean CVs of the dilution groups is statistically significant for the majority of the mutant allele fraction values.

**Table 3:** Data scattering characteristic of the coefficient of variation (CV) values in a batch of samples with mutations

Mutant allele fraction (%)	100 copies/PCR	10,000 copies/PCR	Mann–Whitney test (p)
	MCV±σ_CV_ (%)	M_CV_±σ_CV_ (%)	
1	61.82±27.08	13.85±6.93	**0.008**
2	44.06±13.83	9.13±2.95	**0.008**
3	41.69±10.80	8.45±4.04	**0.008**
5	34.55±14.72	6.92±2.79	**0.008**
10	27.31±18.36	4.67±2.02	0.15
30	20.12±16.79	3.18±1.23	**0.016**

**Figure 2.**
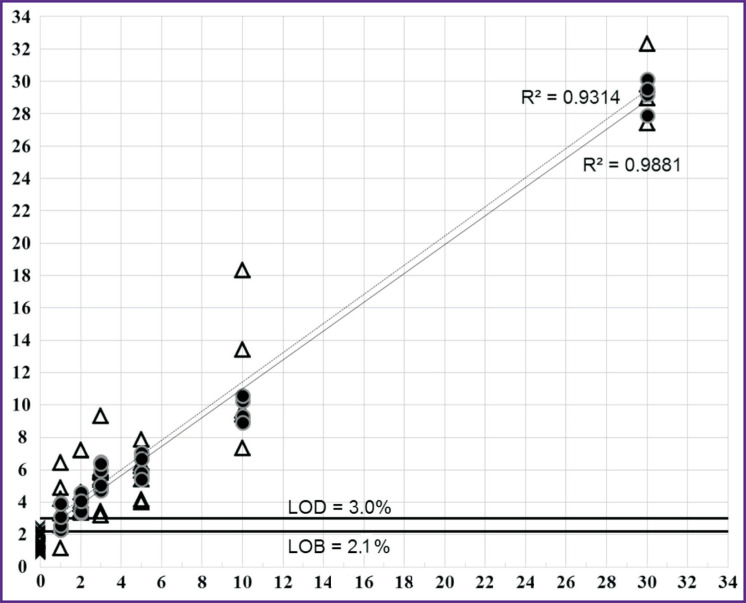
Correlation between the expected and the measured fractions of the mutant allele for the V600E mutation at sequencing with the 600-S primer The X-axis is the expected proportion of the mutant allele in the sample (%); the Y-axis is the measured proportion of the mutant allele (%); crosses are wild samples; circles are dilutions with the V600E mutation, 10,000 copies/PCR; triangles are dilutions with the V600E mutation, 100 copies/PCR. The dotted line shows the trend for 100 copies/PCR, the solid line demonstrates the trend for 10,000 copies/PCR

Thus, a significant spread in values of the measured mutant allele fraction (Figure 2) in samples with the concentration of 100 copies/PCR can reduce the reliability of mutation detection. Based on the data received, the LOD values were calculated for samples with a concentration of 10,000 (see Table 2) and 100 copies/ PCR (no data provided).

### Biological material testing

The concentration of the DNA exported from 132 biological samples ranged from 1.2 to 1128.0 copies/μl. Testing revealed 53 samples with the V600E mutation from 51 patients. The proportion of the mutant allele was 4.9–50.0%. In the test with the BR-S primer, the proportion of the mutant allele in 8 samples was below 10%, which did not allow a precise determination of the mutation type in codon 600. It was not possible to reliably detect a mutation in codon 600 in another sample. Analysis of these samples using the 600-S primer confirmed the V600E mutation in all samples. 77 out of 132 samples were tested using three methods (BR-S, 600-S, and 601-S), 41 — using the BR-S and 601-S primers, 14 — using the BR-S and 600-S primers. There were no discordant results.

The authors also analyzed the paired FNA samples of the thyroid nodule and sentinel lymph node obtained from four patients. In one case, the V600E mutation was found in both samples; the classical variant of papillary thyroid cancer with metastases in 22 of 63 lymph nodes was histologically confirmed. In the second pair, the V600E mutation was also found in both samples at the stage of anaplastic cancer cytological diagnosis. In the third case, the mutation was detected only in the FNA sample of the thyroid nodule; an encapsulated follicular variant of unexpanded papillary thyroid cancer with capsular invasion, which had no metastases in the lymph nodes, was histologically confirmed. In the fourth pair of samples, no mutation was detected; follicular adenoma was histologically confirmed. The V600E mutation was detected only in the FNA sample of the left lobe in another patient with nodules in both lobes of the thyroid gland; histologically, the left lobe was diagnosed with papillary thyroid cancer with multicentric growth, whereas the right lobe — with follicular adenoma of the thyroid gland.

## Discussion

### Analytical characteristics of the methods

In the case of the V600E mutation, when tested using the 600-S primer on 10,000 copies/PCR samples containing 2% of the mutant allele, all measurements were within the range of 3.4–4.6% (3.9±0.5); when testing samples with a concentration of 100 copies/PCR containing 5% of the mutant allele, all measurements were within the range of 4.0–7.9% (5.80±1.26), which allows them to be reliably distinguished from wild samples (see Figure 2). In the V600K and V600R mutations, samples with a high concentration containing 1% of the mutant allele can be determined, whereas for V600M — containing 2% of the mutant allele. In samples with low concentrations, the reliable detection for all mutations starts from 5% of the mutant allele.

In the K601E mutation, when testing samples with a concentration of 10,000 copies/PCR, the 601-S primer ensures the detection of samples containing 3% of the mutant allele. The order of adding nucleotides used for sequencing with the 601-S primer allows the detection of codon 600 mutations, but with a decreased sensitivity: for example, the V600E LOD value was 3.4% for 10,000 copies/PCR.

LOD values for concentrations of 10,000 copies/ PCR were lower compared to those of 100 copies/PCR concentrations. Samples with a low DNA concentration were characterized by highly scattered values of the measured mutant allele fraction, which decreases the likelihood of reliable determination of mutations, which coincides with the previously obtained data [16]. Thus, to increase the reliability of the analysis during biological samples testing, it is recommended to use a high DNA concentration or to test in several replications depending on the DNA concentration.

The results of the study demonstrate the possibility of using pyrosequencing to determine somatic mutations against a significant excess of intact DNA. The newly developed methods increase the mutation detection sensitivity for the K601E mutation from 10% to 3–5% compared to the first version of the method [16].

### Differentiation of codon 600 mutations

The order of adding nucleotides for sequencing using the 600-S primer was chosen so as to allow the determination of nucleotide substitutions in codon 600. Each analyzed mutation corresponded to a unique pattern of the nucleotides signal level changes on the pyrogram (see Figure 1). This allows a precise detection of codon 600 mutations even in a low (below 10%) mutant allele fraction.

A convenient way to differentiate mutations in codon 600 is to determine the ratio of signal levels specific to various mutations. The V600E mutation signal on the pyrogram increases at the T3, C6, and T8 positions, whereas the C2, G4, T5, and C12 level does not exceed the background level fluctuations in wild samples. The V600R mutation signal increases at the C2, T3, G4, T5, and C12 positions; the V600K mutation signal increases at the T3, G4, T5, and C12 positions; the V600M mutation signal increases only for T8 (see Figure 1). Thus, it is sufficient to use combinations of signals in three positions — C2, T3, and T8 to clearly determine mutations (Table 4).

**Table 4:** Determination of mutations using combinations of the C2, T3, and T8 signals

Mutation	Signal value growth on the pyrogram in position		
	C2	T3	T8
V600E	–	+	+
V600K	–	+	–
V600M	–	–	+
V600R	+	+	–

Statistically significant differences for batches of samples with mutations in codon 600 in terms of signal level were established (Kruskal–Wallis test, p<0.0001). The results of the subsequent *post-hoc* analysis of pairwise differences are shown in Table 5. Statistically, each mutation pair significantly differs in at least one parameter, which confirms the possibility of mutations differentiation in codon 600 by analyzing the signal level at three positions of the pyrogram.

**Table 5:** Pairwise comparison of groups with different mutations in codon 600 by signal values in the positions T3, T8, and C2 on pyrograms

Comparison groups	n1/n2	Position	Dunn’s z-score test,	p_adjusted_*
V600E/V600K	46/51	T8	–7.66	**<1·10^–4^**
		T3	2.95	**<0.05**
		C2	–1.07	1.0
V600E/V600M	46/51	T8	1.97	0.3
		T3	–6.75	**<1·10^–4^**
		C2	–3.14	**<0.05**
V600K/V600M	51/51	T8	9.88	**<1·10^–4^**
		T3	–9.96	**<1·10^–4^**
		C2	–2.12	0.2
V600E/V600R	46/48	T8	–6.44	**<1·10^–4^**
		T3	2.12	0.2
		C2	6.94	**<1·10^–4^**
V600K/V600R	51/48	T8	1.13	1.0
		T3	–0.81	1.0
		C2	8.21	**<1·10^–4^**
V600M/V600R	51/48	T8	–8.60	**<1·10^–4^**
		T3	8.99	**<1·10^–4^**
		C2	10.30	**<1·10^–4^**

* is the p-value adjusted for Bonferroni multiple comparisons; n1/n2 is the number of the analyzed samples in the comparison groups.

**Figure 3.**
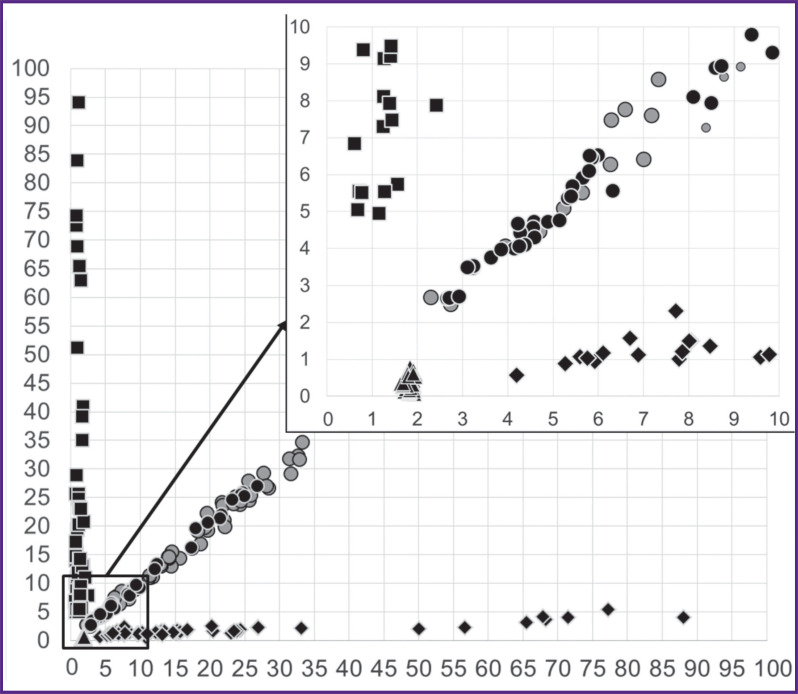
Scattering graph of signal ratios in T3/C2 and T8/C2 positions on pyrograms for samples with mutations in codon 600 The X-axis is the ratio of signals in the T3/C2 positions; the Y-axis is the ratio of signals in the T8/C2 positions of the pyrogram; triangles are dilutions with the V600R mutation, rhombuses are dilutions with the V600K mutation, squares are dilutions with the V600M mutation, dark circles are dilutions with the V600E mutation, light circles are samples of thyroid nodules with the V600E mutation

Taking into account that three independent variables form a three-dimensional space, the convenience of graphical representation was achieved by reducing the dimension through conversion of three variables into two ratios — T3/C2 and T8/C2. Samples with codon 600 mutations according to the T3/C2 and T8/C2 signals ratios were clustered into four non-intersecting groups. This allowed differentiating the V600E, V600K, V600R, and V600M mutations by the ratio of T3/C2 and T8/C2 peaks for samples containing the mutant allele fraction over the LOD value for the corresponding mutations (Figure 3).

Validation of the developed complex of methods on samples of biological material proved its effectiveness for identification and determination of the *BRAF* mutation types even in samples with a low (less than 500 copies/ PCR) DNA concentration and a low (less than 10%) mutant allele fraction. The authors established a significant correlation between the mutant allele fractions obtained by different methods: the Pearson correlation coefficient amounted to 0.99 (95% CI 0.98–0.99; p<0.001) for 600-S and 601-S; 0.96 (95% CI 0.92–0.98; p<0.001) for BR-S and 601-S; 0.96 (95% CI 0.93–0.98; p<0.001) for BR-S and 600-S. The use of an additional sequencing primer (600-S) provided for precise determination of the V600E mutation in 9 samples with the proportion of the mutant allele below 10%.

Thus, the proposed complex of methods to determine mutations in the *BRAF* codons 592–602, to differentiate mutations in codon 600 and detect the K601E mutation, as well as the algorithm for the pyrosequencing results’ interpretation, allow to increase the range of detected mutations and improve sensitivity and specificity compared to previously proposed methods [12-14, 16, 21].

### Biological material testing

As of the time of analysis, 42 out of 51 patients with the V600E mutation had papillary thyroid cancer confirmed (28 were diagnosed histologically), 6/51 had suspicious for malignancy (Bethesda V), and 1/51 patients had anaplastic thyroid cancer (Bethesda VI); there were also the following cases identified: 1/51 had atypia of undetermined significance (Bethesda III), and 1/51 had no established diagnosis.

In a study of 128 samples from 127 patients, mutations were detected in 42 of 53 persons diagnosed with papillary thyroid cancer (in 38 patients, the diagnosis was histologically confirmed), 6/13 had suspicious for malignancy (Bethesda V), 1/10 had atypia of undetermined significance (Bethesda III), one patient had anaplastic cancer (Bethesda VI), and 1/7 had no established diagnosis. In 6 patients with benign lesions (5 had adenomatous goiter, 1 had histologically identified multinodular goiter), 1 NIFTP patient and 37 patients with follicular tumors (Bethesda IV, 17 were histologically identified), no mutations were determined. The results of the determination of the mutation in 4 pairs of samples of the thyroid nodule and lymph node for all patients were consistent with histopathology reports. A higher frequency of the V600E mutation (in 42 of 53 patients, 79%) compared to the incidence described in other papillary cancer studies [1, 3, 4, 9, 11] was due to the fact that a group of samples with the V600E mutation, which was previously determined using the first version of the method, was included to validate new methods [16].

## Conclusion

The complex of pyrosequencing-based methods for determining the nucleotide sequence of the *BRAF* 592–601 codons and the algorithm for sample testing and mutation analysis in the *BRAF* codons 600–601 were developed. The new methods allow a definite differentiation of all tested mutations in case of a low proportion of the mutant allele and an increase in the sensitivity of the assay to 1–5% of the mutant allele compared to the assay with a single sequencing primer.

When testing biological samples, 53 samples with the V600E mutation were detected, and the proportion of the mutant allele was 4.9–50.0%. The results obtained using different primers were similar for all samples. The use of additional sequencing primers (600-S, 601-S) allows the determination of the detected mutations types with the mutant allele fraction below 10%.

The proposed approach allows the development of similar methods to identify rare mutations in a sequenced fragment and mutations in other oncogenes (*K-*, *H-*, *N-RAS*).
